# Suppression of Coronavirus Replication by Cyclophilin Inhibitors

**DOI:** 10.3390/v5051250

**Published:** 2013-05-22

**Authors:** Yoshikazu Tanaka, Yuka Sato, Takashi Sasaki

**Affiliations:** 1 Department of Veterinary Hygiene, Veterinary School, Nippon Veterinary & Life Science University, Tokyo 180-8602, Japan; 2 Department of Microbiology and Immunology, Division of Molecular Virology, The Institute of Medical Science, The University of Tokyo, Tokyo 108-8639, Japan; 3 Laboratory of Bacterial Genomics, Pathogen Genomics Center, National Institute of Infectious Diseases, Tokyo 162-8640, Japan

**Keywords:** cyclophilin, cyclosporin A, coronavirus, NF-AT

## Abstract

Coronaviruses infect a variety of mammalian and avian species and cause serious diseases in humans, cats, mice, and birds in the form of severe acute respiratory syndrome (SARS), feline infectious peritonitis (FIP), mouse hepatitis, and avian infectious bronchitis, respectively. No effective vaccine or treatment has been developed for SARS-coronavirus or FIP virus, both of which cause lethal diseases. It has been reported that a cyclophilin inhibitor, cyclosporin A (CsA), could inhibit the replication of coronaviruses. CsA is a well-known immunosuppressive drug that binds to cellular cyclophilins to inhibit calcineurin, a calcium-calmodulin-activated serine/threonine-specific phosphatase. The inhibition of calcineurin blocks the translocation of nuclear factor of activated T cells from the cytosol into the nucleus, thus preventing the transcription of genes encoding cytokines such as interleukin-2. Cyclophilins are peptidyl-prolyl isomerases with physiological functions that have been described for many years to include chaperone and foldase activities. Also, many viruses require cyclophilins for replication; these include human immunodeficiency virus, vesicular stomatitis virus, and hepatitis C virus. However, the molecular mechanisms leading to the suppression of viral replication differ for different viruses. This review describes the suppressive effects of CsA on coronavirus replication.

## 1. Introduction

Coronaviruses (CoVs) infect a variety of mammalian and avian species and cause serious diseases in humans, cats, mice, and birds in the form of severe acute respiratory syndrome (SARS), feline infectious peritonitis (FIP), mouse hepatitis, and avian infectious bronchitis, respectively. The 2002/2003 outbreak of SARS has demonstrated human vulnerability to CoV epidemics [1]. CoVs are members of the *Coronaviridae* family; they contain a large positive-sense single-stranded RNA genome of approximately 30 kb in length, and express several structural proteins, including the spike (S), envelope (E), membrane (M), and nucleocapsid (N) proteins. In addition to these structural proteins, many non-structural proteins (nsp) are also expressed. In *Coronaviridae*, two-thirds of the genome is found within the 5'-genomic region that contains open-reading frame 1a (ORF1a) and open-reading frame 1b (ORF1b); these encode RNA-dependent RNA polymerase (RdRp), helicase, and other nsp proteins. ORF1a and ORF1b are initially synthesized as two large polyproteins, pp1a and pp1ab. The synthesis of pp1ab involves programmed ribosomal frame shifting during translation of ORF1a [2]. These proteins are cleaved by virus-encoded proteinases. ORF1a encodes proteins nsp1 to nsp11 and ORF1b encodes proteins nsp12 to nsp16. In the remaining third of the genome, genes for the structural proteins are encoded in a fixed order: S-E-M-N. A variable number of ORF encoding accessory proteins are present between these genes [3]. 

Cyclosporin A (CsA) is a well-known immunosuppressive drug that binds to cellular cyclophilins (Cyps) to inhibit calcineurin, a calcium-calmodulin-activated serine/threonine-specific phosphatase. The inhibition of calcineurin blocks the translocation of nuclear factor of activated T cells (NF-AT) from the cytosol into the nucleus, thus preventing the transcription of genes encoding cytokines such as interleukin-2 [4]. There are seven major Cyps in humans-cyclophilin A (CypA), cyclophilin B (CypB), cyclophilin C (CypC), cyclophilin D (CypD), cyclophilin E (CypE), cyclophilin 40 (Cyp40), and cyclophilin NK (CypNK). They are generally not linked to each other in the genome [5]. Cyps are peptidyl–prolyl isomerases (PPIases) with physiological functions that have been described for many years as including chaperone and foldase activities [6]. Cyps are thought to be involved in diverse signaling pathways, including mitochondrial apoptosis [7,8], RNA splicing [9,10], and adaptive immunity [11]. However, the proteins that act as substrates for Cyps in these pathways have not yet been identified.

Recently, several researchers have shown that CypA is involved in the pathogenesis of viral infection [12], cardiovascular diseases [13], and cancer [14]. CypA plays a critical role in the replication of viruses such as human immunodeficiency virus type 1 (HIV-1) [15], hepatitis C virus (HCV) [16], vesicular stomatitis virus (VSV) [17], human papilloma virus [18], and vaccinia virus [19]. Therefore, CsA and non-immunosuppressive compounds that bind to Cyps such as NIM811, Debio-025, and SCY-635 inhibit HIV-1 and HCV replication [20,21,22,23,24]. Also, several laboratories have reported that CsA suppresses the replication of various kinds of CoVs [25,26,27].

In this review, we focused on the relationship between CsA and the replication of CoVs, and introduce some of the recent advances in this area.

## 2. CsA Inhibits the Replication of Feline CoV

CoV replication relies on a variety of host factors, which also constitute potentially interesting targets for antiviral therapy [28]. Feline CoV (FCoV) is classified into two biotypes, the ubiquitous feline enteric CoV (FECV) and infectious peritonitis virus (FIPV). The widely accepted theory from *in vitro* studies is that FIPV arises by mutation from parental FECV in the gastrointestinal tract of infected cats [29,30]. Many strategies for curing FIP have been attempted. Interferon ω inhibits FIPV *in vitro*, but is ineffective *in vivo* [28]. Various other immunosuppressants, such as glucocorticoids and cyclophosphamide, have also been investigated; however, although these agents can prolong life, the outcome of FIPV infection remains fatal [31]. Thus, an effective vaccine and therapeutic medicine against FIPV are still needed.

We discovered that replication of FCoV was inhibited by CsA in a dose-dependent manner [32]. CsA binds to cellular Cyps to block the NF-AT pathway; therefore, we tried using an immunosuppressive agent, FK506, which binds to FK506 binding protein (FKBP), to block the NF-AT pathway. FK506, however, had no effect on FCoV replication and translation. This result indicates that the inhibition effect of CsA on FCoV does not involve the NF-AT pathway and its related immunosuppressive effects. We then examined whether the suppressive effects of CsA on FIPV replication depended on the P-glycoprotein pathway by incubating FIPV-infected cells with cyclosporin H (CsH), a P-glycoprotein pathway-specific inhibitor; however, no inhibition occurred. To determine whether the effects of CsA and FK506 involve the activation of interferon-stimulated gene responses in fcwf-4 cells, an interferon-stimulated response element (ISRE)-luciferase reporter assay was performed. However, neither interferon α stimulation nor treatment with CsA and FK506 had any effect on ISRE-promoter activities in fcwf-4 cells [27]. Therefore, other roles of Cyps appear to be required for viral replication.

## 3. CsA Inhibits the Replication of Diverse CoVs

De Wilde *et al*. reported that CsA inhibited the replication of CoVs (SARS–CoV, human 229E CoV, and mouse hepatitis virus (MHV)-A59) using green fluorescence protein (GFP)-expressing recombinant CoVs [25]. Namely, they used Vero-E6 cells and 293/ACE2 cells (these cells stably express the SARS–CoV receptor, ACE2) for infection of SARS–CoV. To investigate whether CsA inhibits the replication of other CoVs, Huh7 cells infected with HCoV–229E–GFP and 17CL1 cells infected with MHV–GFP were treated with CsA. GFP expression decreased in all infected cells, although to a lesser degree in HCoV–229E–GFP-infected cells. Remarkably, approximately 1% to 5% of the infected cells remained SARS–CoV positive by immunofluorescence analysis, even at CsA concentrations of up to 64 µM. These results suggest that SARS–CoV replication was indeed impaired, although not fully blocked. The used virus may have been one of the naturally CsA-resistant viruses that are sometimes present in the virus pool. Several other RNA viruses require CypA or CypB in the replication process, as has been described for HCV, equine arterivirus (EAV), Japanese encephalitis virus, VSV, and West Nile virus (WNV) [16,17,33,34,35]. Therefore, de Wilde *et al*. also examined whether CsA might exert an inhibitory effect on CoV replication by inhibiting Cyp function and by indirect inhibition of virus-specific function; however, they found that the activity of nsp12, an RdRp in SARS–CoV, was not directly affected. In addition, depletion of cellular CypA or CypB by knockdown experiments did not significantly affect SARS–CoV replication; however, it is possible that remaining Cyp in the small interference RNA (siRNA)-treated cells was sufficient to support normal virus replication.

## 4. Nsp1 Protein Increases Signaling Through the Calcineurin/NF-AT Pathway

SARS–CoV Nsp1 is an *in vivo* virulence factor whose action has been linked to the early stages of the immune response, including antagonistic activity against interferon signaling and inhibition of host protein synthesis [36,37]. Pfefferle *et al*. performed a genome-wide yeast two-hybrid interaction screen with the amino (N)-terminal region of the Nsp1 protein and identified the proteins that acted as interactive partners (CypA, CypB, CypH, CypG, FKBP1A, FKBP1B) [26]. These molecules are known to function as regulators of the NF-AT pathway and play an important role in the immune system. Overexpression of Nsp1 or infection with SARS–CoV strongly increases signaling through the NF-AT pathway and enhances the induction of interleukin-2 in the presence of phorbol 12-myristate 13-acetate (PMA) and ionomycin. These results suggest that the NF-AT signaling pathway has an influence on the fundamental triggers of immune cell activation, providing an explanation for the cytokine dysregulation and immune-dependent pathogenesis observed in severe cases of SARS. In addition, Pfefferle *et al*. found that CsA inhibited the replication of other CoV members, including human CoV–NL63, 229E, FCoV (serotypes I and II), SARS–CoV, porcine transmissible gastroenteritis virus (TGEV), and avian infectious bronchitis virus (IBV). Evidence shows that CsA binds to intracellular Cyps and blocks the NF-AT pathway. However, it appears that the inhibition effects of CsA on virus replication are independent from the NF-AT pathway. Although Nsp1 interacted with FKBP1A and FKBP1B, Pfefferle *et al*. did not examine whether FK506 inhibited the replication of CoV. In addition, we have reported that FK506 does not affect the replication of FCoV [27]. Moreover, Brockway and Denison showed that deletion of residues in the amino-terminal half of nsp1 blocked productive infection of the virus [38]. Since recombinant viruses with a deletion in the N-terminal half of nsp1 do not appear to bind to Cyps, it is difficult to elucidate the exact role of nsp1 in virus–host interactions. The Cyp–CsA complex may inhibit the other functions of Cyps that are required for virus replication.

## 5. Cyp Inhibitors Block Arterivirus Replication

Using GFP-expressing recombinant equine arteritis virus (EAV) and porcine reproductive and respiratory syndrome virus (PRRSV), de Wilde *et al*. recently reported that CsA inhibits arteriviruses, which are distantly related to CoVs; together with the *Coronaviridae* and *Roniviridae* families, they constitute the order *Nidovirales* [33]. CsA as an immunosuppressive agent and Debio-064 as a non-immunosuppressive agent inhibited EAV and PRRSV replication. CsA strongly reduced EAV progeny titers, with an almost 4-log-unit reduction at 4 µM CsA. These data correlated well with the barely detectable expression levels of the nsp5–8, nsp9, M, and N proteins after treatment with 4 µM CsA. Moreover, treatment with Debio-064 also resulted in an approximately 4-log-unit reduction of infectious progeny at 2 µM CsA, while a 2- to 3-log-unit reduction was achieved by treatment with only 1 µM of the compound. Compared to the effects on EAV, significantly higher concentrations of CsA were required to completely block the infectious progeny of PRRSV (32 µM CsA was required to achieve a 2.5-log-unit reduction). Even treatment with Debio-064 resulted in only an approximately 1.5-log-unit reduction at 16 µM and an approximately 2.5-log-unit reduction at 32 µM. Debio-064 possesses a higher affinity for Cyps than CsA, as seen from the results of the EAV experiments. However, the concentration required to inhibit virus replication is different for each virus. The required concentration may also be affected by the use of different cell lines in replication experiments.

## 6. EAV Replication Depends on CypA

De Wilde *et al*. also reported that EAV replication was inhibited by knockdown of CypA expression, but not by knockdown of CypB expression [33]. The titers of virus progeny decreased approximately fourfold in CypA-knockdown cells as compared to those in the non-targeted cells. These experiments were performed using transient expression of siRNAs against the Cyps. Although an approximately 80% reduction in Cyp expression level was achieved, the residual Cyp expression (approximately 20%) that remained after knockdown may have been sufficient for normal virus replication. However, compared to the inhibition effects of CsA on the GFP-expressing recombinant virus, the inhibition effects of CypA knockdown are much lower. Consequently, other mechanisms for the effect of CypA on virus replication through direct and indirect interactions with other cellular factors or through other signaling pathways cannot be excluded.

## 7. Conclusions

CsA can inhibit the replication of various kinds of RNA viruses, including HIV-1, HCV, and several flaviviruses [35,39]. There have been more published reports on HCV replication than for any other virus. HCV replication is affected by multiple Cyps. The PPIase activity of Cyps plays a role in the assembly of the HCV viral replicase complex (VRC); in particular, the cytosolic CypA has been shown to be critical for HCV VRC assembly, likely by affecting HCV polyprotein processing, NS5A–NS5B fusion protein cleavage, and the NS5A and NS5B RdRp [40,41]. In addition, CypA-independent NS5A mutants showed that CypA is likely involved in the folding of a non-structured region in NS5A, which may require a structural shift towards a more extended conformation during HCV replication [42]. However, direct physical interactions between RdRp of the CoVs and Cyps have not been reported.

CD147 (EMMPRIN), a type I transmembrane protein, has been identified as the main signaling receptor for extracellular CypA [43]. Although none of the SARS–CoV proteins directly bound to CD147, the N protein of SARS–CoV bound to CypA, which interacted with CD147, by surface plasmon resonance analysis. CD147 antagonist showed a high rate of binding to 293 cells and an inhibitory effect on SARS–CoV. These results demonstrate that CD147 plays a functional role in facilitating SARS–CoV invasion into host cells [44]. CsA inhibition of CypA function may suppress the production of SARS–CoV progeny.

Cyps may affect the conformation of the viral RdRp or the RNA template, which in turn could alter the template activity of the RdRp [16,17]. The N protein of VSV interacts with CypA. When CypA is incorporated into the virus particle, it appears to act as a chaperone for the N protein by wrapping genomic RNA to produce a functional template for transcription. Also, CypB, a resident protein in the endoplasmic reticulum (ER)-Golgi, was found to regulate the RNA-binding ability of the HCV NS5B RdRp [16]. Other ER-Golgi resident proteins such as CypC also affect trafficking of the viral proteins [45]. If the N protein of *Coronaviridae* family viruses such as SARS–CoV binds to Cyps, it is possible that Cyps act as a chaperone for the N protein and play an important role in replication.

CD147–CypA interaction plays a critical role in HIV-1 infection [46]. The CD147–CypA complex could induce phosphorylation of the matrix protein (MA) to regulate the detachment of the reverse transcriptase complex from the membrane or promote transition from the step of hemifusion. Moreover, it is proposed that CD147–CypA interaction might indirectly affect capsid conformation. Efficient incorporation of CypA into the HIV-1 virion is mediated by a direct prolyl peptide bond between the CypA and a proline-rich loop in the HIV-1 capsid protein [47]. Disruption of CypA incorporation by Gag mutations or by treatment with CsA attenuated the infectivity of progeny viruses [48]. However, there is no evidence that the CD147–CypA complex affects the step of fusion in CoVs.

**Figure 1:**
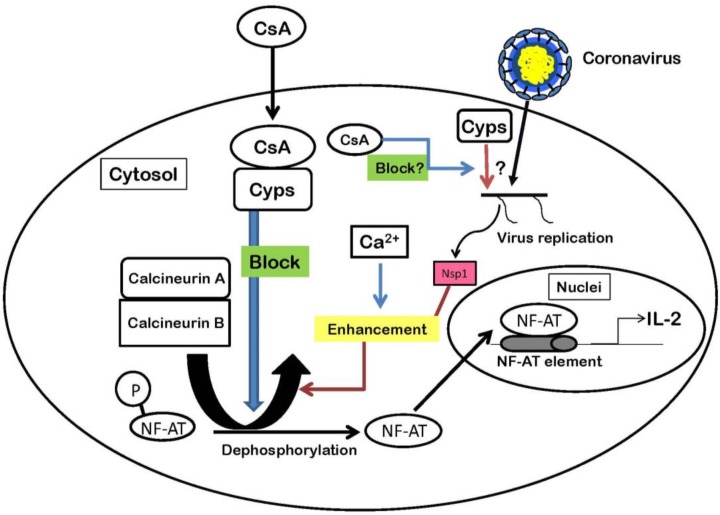
The functions of Cyps in the NF-AT pathway and a hypothesis of the interaction between Cyps and coronavirus. Nsp1 protein enhances NF-AT activities with Ca^2+^. The activities result in production of IL-2. Although CsA inhibits CoV replication, little is known about the exact role of Cyps in CoV replication.

CsA inhibits *Nidovirales* replication *in vitro*. Although depletion of cellular CypA by knockdown experiments inhibits virus replication of EAV, those of SARS–CoV is not affected (Table 1). Also, we showed the roles of Cyps in CoV replication (Figure 1). Little is known about the exact role of CypA in CoV replication and how CsA inhibits virus replication by binding to Cyps. Proteomics approaches and structural studies will provide more detailed information on the role of Cyps in regulating the composition and structure of viral protein complexes. Cyclosporins such as CsA and its analogs, Debio-025, NIM811 and SCY-635, target cellular Cyps as described above. Thus, viruses resistant to cyclosporins are unlikely to develop, because antiviral compounds targeting cellular factors generally produce less drug resistance than those targeting viral proteins [49]. Therefore, cyclosporins are good candidates for therapeutic medicines against CoV and other RNA virus infections.

**Table 1:** Inhibition effects of virus replication by CsA treatment and knockdown Cyps.

			Inhibition of virus replication by CsA treatment	Knockdown CypA	Knockdown CypB	Ref.
				Virus replication		
*Nidovirales*	*Coronaviridae*	SARS CoV	+	Not affected	Not affected	[25]
		HCoV–NL63	+	Unknown	Unknown	[26]
		HCoV–229E	+	Unknown	Unknown	[26]
		TGEV	+	Unknown	Unknown	[26]
		IBV	+	Unknown	Unknown	[26]
		MHV	+	Unknown	Unknown	[25]
		FCoV	+	Unknown	Unknown	[26]
	*Arteriviridae*	EAV	+	Inhibition of virus replication	Not affected	[33]
		PRRSV	+	Unknown	Unknown	[33]

## References

[1] Perlman S., Netland J. (2009). Coronaviruses post-sars: Update on replication and pathogenesis. Nat. Rev. Microbiol..

[2] Namy O., Moran S.J., Stuart D.I., Gilbert R.J., Brierley I. (2006). A mechanical explanation of RNA pseudoknot function in programmed ribosomal frameshifting. Nature.

[3] Sawicki S.G., Sawicki D.L., Siddell S.G. (2007). A contemporary view of coronavirus transcription. J. Virol..

[4] Quesniaux V.F., Schreier M.H., Wenger R.M., Hiestand P.C., Harding M.W., van Regenmortel M.H. (1987). Cyclophilin binds to the region of cyclosporine involved in its immunosuppressive activity. Eur. J. Immunol..

[5] Wang P., Heitman J. (2005). The cyclophilins. Genome Biol..

[6] Gething M.J., Sambrook J. (1992). Protein folding in the cell. Nature.

[7] Leung A.W., Halestrap A.P. (2008). Recent progress in elucidating the molecular mechanism of the mitochondrial permeability transition pore. Biochim. Biophys. Acta.

[8] Leung A.W., Varanyuwatana P., Halestrap A.P. (2008). The mitochondrial phosphate carrier interacts with cyclophilin D and may play a key role in the permeability transition. J. Biol. Chem..

[9] Dubourg B., Kamphausen T., Weiwad M., Jahreis G., Feunteun J., Fischer G., Modjtahedi N. (2004). The human nuclear srcyp is a cell cycle-regulated cyclophilin. J. Biol. Chem..

[10] Teigelkamp S., Achsel T., Mundt C., Gothel S.F., Cronshagen U., Lane W.S., Marahiel M., Luhrmann R. (1998). The 20kD protein of human [U4/U6.U5] tri-snRNPs is a novel cyclophilin that forms a complex with the U4/U6-specific 60kD and 90kD proteins. RNA.

[11] Anderson S.K., Gallinger S., Roder J., Frey J., Young H.A., Ortaldo J.R. (1993). A cyclophilin-related protein involved in the function of natural killer cells. Proc. Natl. Acad. Sci. USA.

[12] Zhou D., Mei Q., Li J., He H. (2012). Cyclophilin A and viral infections. Biochem. Biophys. Res. Commun..

[13] Satoh K., Shimokawa H., Berk B.C. (2010). Cyclophilin A: Promising new target in cardiovascular therapy. Circ. J..

[14] Obchoei S., Wongkhan S., Wongkham C., Li M., Yao Q., Chen C. (2009). Cyclophilin A: Potential functions and therapeutic target for human cancer. Med. Sci. Monit..

[15] Luban J., Bossolt K.L., Franke E.K., Kalpana G.V., Goff S.P. (1993). Human immunodeficiency virus type 1 Gag protein binds to cyclophilins A and B. Cell.

[16] Watashi K., Ishii N., Hijikata M., Inoue D., Murata T., Miyanari Y., Shimotohno K. (2005). Cyclophilin B is a functional regulator of hepatitis C virus RNA polymerase. Mol. Cell.

[17] Bose S., Mathur M., Bates P., Joshi N., Banerjee A.K. (2003). Requirement for cyclophilin a for the replication of vesicular stomatitis virus new jersey serotype. J. Gen. Virol..

[18] Bienkowska-Haba M., Patel H.D., Sapp M. (2009). Target cell cyclophilins facilitate human papillomavirus type 16 infection. PLoS Pathog..

[19] Castro A.P., Carvalho T.M., Moussatche N., Damaso C.R. (2003). Redistribution of cyclophilin a to viral factories during vaccinia virus infection and its incorporation into mature particles. J. Virol..

[20] Billich A., Hammerschmid F., Peichl P., Wenger R., Zenke G., Quesniaux V., Rosenwirth B. (1995). Mode of action of SDZ NIM 811, a nonimmunosuppressive cyclosporin A analog with activity against human immunodeficiency virus (HIV) type 1: Interference with hiv protein-cyclophilin a interactions. J. Virol..

[21] Ptak R.G., Gallay P.A., Jochmans D., Halestrap A.P., Ruegg U.T., Pallansch L.A., Bobardt M.D., de Bethune M.P., Neyts J., de Clercq E. (2008). Inhibition of human immunodeficiency virus type 1 replication in human cells by Debio-025, a novel cyclophilin binding agent. Antimicrob. Agents Chemother..

[22] Goto K., Watashi K., Murata T., Hishiki T., Hijikata M., Shimotohno K. (2006). Evaluation of the anti-hepatitis C virus effects of cyclophilin inhibitors, cyclosporin A, and NIM811. Biochem. Biophys. Res. Commun..

[23] Hopkins S., Scorneaux B., Huang Z., Murray M.G., Wring S., Smitley C., Harris R., Erdmann F., Fischer G., Ribeill Y. (2010). Scy-635, a novel nonimmunosuppressive analog of cyclosporine that exhibits potent inhibition of hepatitis C virus RNA replication *in vitro*. Antimicrob. Agents Chemother..

[24] Paeshuyse J., Kaul A., de Clercq E., Rosenwirth B., Dumont J.M., Scalfaro P., Bartenschlager R., Neyts J. (2006). The non-immunosuppressive cyclosporin Debio-025 is a potent inhibitor of hepatitis C virus replication *in vitro*. Hepatology.

[25] de Wilde A.H., Zevenhoven-Dobbe J.C., van der Meer Y., Thiel V., Narayanan K., Makino S., Snijder E.J., van Hemert M.J. (2011). Cyclosporin a inhibits the replication of diverse coronaviruses. J. Gen. Virol..

[26] Pfefferle S., Schopf J., Kogl M., Friedel C.C., Muller M.A., Carbajo-Lozoya J., Stellberger T., von Dall’Armi E., Herzog P., Kallies S. (2011). The SARS-coronavirus-host interactome: Identification of cyclophilins as target for pan-coronavirus inhibitors. PLoS Pathog..

[27] Tanaka Y., Sato Y., Osawa S., Inoue M., Tanaka S., Sasaki T. (2012). Suppression of feline coronavirus replication *in vitro* by cyclosporin A. Vet. Res..

[28] Pedersen N.C. (2009). A review of feline infectious peritonitis virus infection: 1963–2008. J. Feline Med. Surg..

[29] Pedersen N.C., Boyle J.F., Floyd K., Fudge A., Barker J. (1981). An enteric coronavirus infection of cats and its relationship to feline infectious peritonitis. Am. J. Vet. Res..

[30] Rottier P.J., Nakamura K., Schellen P., Volders H., Haijema B.J. (2005). Acquisition of macrophage tropism during the pathogenesis of feline infectious peritonitis is determined by mutations in the feline coronavirus spike protein. J. Virol..

[31] Hartmann K., Ritz S. (2008). Treatment of cats with feline infectious peritonitis. Vet. Immunol. Immunopathol..

[32] Tanaka Y., Osawa S., Inoue M., Tanaka S., Sato Y. Inhibition of Feline Infectious Peritonitis Viral Replication by Cyclosporine. Proceedings of the 29th Annual Meeting of the American Society for Virology.

[33] de Wilde A.H., Li Y., van der Meer Y., Vuagniaux G., Lysek R., Fang Y., Snijder E.J., van Hemert M.J. (2013). Cyclophilin inhibitors block arterivirus replication by interfering with viral RNA synthesis. J. Virol..

[34] Kambara H., Tani H., Mori Y., Abe T., Katoh H., Fukuhara T., Taguwa S., Moriishi K., Matsuura Y. (2011). Involvement of cyclophilin B in the replication of Japanese encephalitis virus. Virology.

[35] Qing M., Yang F., Zhang B., Zou G., Robida J.M., Yuan Z., Tang H., Shi P.Y. (2009). Cyclosporine inhibits flavivirus replication through blocking the interaction between host cyclophilins and viral NS5 protein. Antimicrob. Agents Chemother..

[36] Wathelet M.G., Orr M., Frieman M.B., Baric R.S. (2007). Severe acute respiratory syndrome coronavirus evades antiviral signaling: Role of nsp1 and rational design of an attenuated strain. J. Virol..

[37] Zust R., Cervantes-Barragan L., Kuri T., Blakqori G., Weber F., Ludewig B., Thiel V. (2007). Coronavirus non-structural protein 1 is a major pathogenicity factor: Implications for the rational design of coronavirus vaccines. PLoS Pathog..

[38] Brockway S.M., Denison M.R. (2005). Mutagenesis of the murine hepatitis virus nsp1-coding region identifies residues important for protein processing, viral RNA synthesis, and viral replication. Virology.

[39] Nagy P.D., Wang R.Y., Pogany J., Hafren A., Makinen K. (2011). Emerging picture of host chaperone and cyclophilin roles in RNA virus replication. Virology.

[40] Coelmont L., Hanoulle X., Chatterji U., Berger C., Snoeck J., Bobardt M., Lim P., Vliegen I., Paeshuyse J., Vuagniaux G. (2010). DEB025 (Alisporivir) inhibits hepatitis C virus replication by preventing a cyclophilin a induced cis-trans isomerisation in domain II of NS5A. PLoS One.

[41] Kaul A., Stauffer S., Berger C., Pertel T., Schmitt J., Kallis S., Zayas M., Lohmann V., Luban J., Bartenschlager R. (2009). Essential role of cyclophilin A for hepatitis C virus replication and virus production and possible link to polyprotein cleavage kinetics. PLoS Pathog..

[42] Yang F., Robotham J.M., Grise H., Frausto S., Madan V., Zayas M., Bartenschlager R., Robinson M., Greenstein A.E., Nag A. (2010). A major determinant of cyclophilin dependence and cyclosporine susceptibility of hepatitis C virus identified by a genetic approach. PLoS Pathog..

[43] Yurchenko V., Zybarth G., O’Connor M., Dai W.W., Franchin G., Hao T., Guo H., Hung H.C., Toole B., Gallay P. (2002). Active site residues of cyclophilin A are crucial for its signaling activity via CD147. J. Biol. Chem..

[44] Chen Z., Mi L., Xu J., Yu J., Wang X., Jiang J., Xing J., Shang P., Qian A., Li Y. (2005). Function of HAb18G/CD147 in invasion of host cells by severe acute respiratory syndrome coronavirus. J. Infect. Dis..

[45] Gaither L.A., Borawski J., Anderson L.J., Balabanis K.A., Devay P., Joberty G., Rau C., Schirle M., Bouwmeester T., Mickanin C. (2010). Multiple cyclophilins involved in different cellular pathways mediate HCV replication. Virology.

[46] Pushkarsky T., Zybarth G., Dubrovsky L., Yurchenko V., Tang H., Guo H., Toole B., Sherry B., Bukrinsky M. (2001). CD147 facilitates HIV-1 infection by interacting with virus-associated cyclophilin A. Proc. Natl. Acad. Sci. USA.

[47] An P., Wang L.H., Hutcheson-Dilks H., Nelson G., Donfield S., Goedert J.J., Rinaldo C.R., Buchbinder S., Kirk G.D., O’Brien S.J. (2007). Regulatory polymorphisms in the cyclophilin A gene, PPIA, accelerate progression to AIDS. PLoS Pathog..

[48] Takeuchi H. (2010). Contribution of cyclophilin A to determination of simian immunodeficiency virus tropism: A progress update. Vaccine.

[49] Provencher V.M., Coccaro E., Lacasse J.J., Schang L.M. (2004). Antiviral drugs that target cellular proteins may play major roles in combating hiv resistance. Curr. Pharm. Des..

